# Adipose-derived mesenchymal stem cells rescue rat hippocampal cells from aluminum oxide nanoparticle-induced apoptosis *via* regulation of P53, Aβ, SOX2, OCT4, and CYP2E1

**DOI:** 10.1016/j.toxrep.2021.06.003

**Published:** 2021-06-03

**Authors:** Mona M. Atia, Alshaimaa A.I. Alghriany

**Affiliations:** Laboratory of Molecular Cell Biology, Department of Zoology, Faculty of Science, Assiut University, Egypt

**Keywords:** Al_2_O_3_-NPs, Aluminum oxide nanoparticles, ROS, reactive oxygen species, AD-MSCs, adipose-derived mesenchymal stem cells, Aβ, amyloid beta, EGTA, ethylene glycol tetraacetic acid, TEM, transmission electron microscopy, Sox2, sex-determining region Y-box 2, Oct4, octamer-binding transcription factor 4, MAO-A and B, monoamine oxidase A, B, Aluminum oxide nanoparticles, Adipose-Derived mesenchymal stem cells, Hippocampal cells, Apoptosis

## Abstract

Mesenchymal stem cells (MSCs) possess a preventive capacity against free radical toxicity in various tissues. The present study aimed to demonstrate the reformative and treatment roles of adipose-derived MSCs (AD-MSCs) against severe toxicity in the hippocampal cells of the brain caused by aluminum oxide nanoparticles (Al_2_O_3_-NPs). Rats were divided into five experimental groups: an untreated control group, a control group receiving NaCl, a group receiving Al_2_O_3_-NPs (6 mg/kg) for 20 days, a group that was allowed to recover (R) for 20 days following treatment with Al_2_O_3_-NPs, and a Al_2_O_3_-NPs + AD-MSCs group, where each rat was injected with 0.8 × 10^6^ AD-MSCs *via* the caudal vein. Oral administration of Al_2_O_3_-NPs increased the protein levels of P53, cleaved caspase-3, CYP2E1, and beta-amyloid (Aβ); contrarily, AD-MSCs transplantation downregulated the levels of these proteins. In addition, the AD-MSCs-treated hippocampal cells were protected from Al_2_O_3_-NPs-induced toxicity, as detected by the expression levels of Sox2 and Oct4 that are essential for the maintenance of self-renewal. It was also found that AD-MSCs injection significantly altered the levels of brain total peroxide and monoamine oxidase (MAO)-A and MAO-B activities. Histologically, our results indicated that AD-MSCs alleviated the severe damage in the hippocampal cells induced by Al_2_O_3_-NPs. Moreover, the role of AD-MSCs in reducing hippocampal cell death was reinforced by the regulation of P53, cleaved caspase-3, Aβ, and CYP2E1 proteins, as well as by the regulation of SOX2 and OCT4 levels and MAO-A and MAO-B activities.

## Highlights

• Al_2_O_3_-NPs induce severe toxicity and apoptosis in the hippocampal cells.
• Al_2_O_3_-NPs Stimulated of oxidative stress and increase in Aβ protein levels that activating pro-apoptotic pathway via cleaved caspase-3, P53, and P450.
• Al_2_O_3_-NPs increasing the levels of total peroxide and the activities of MAO-A and MAO-B as well as decreasing the expression of Sox2 and Oct4 proteins.
• AD-MSCs suppress oxidative stress and stimulate immunity when rat are exposed to Al_2_O_3_-NPs.
• AD-MSCs alleviate the toxicity action of Al_2_O_3_-NPs via regulation of P53, Aβ, SOX2, OCT4, and CYP2E1signaling in a hippocampal cells.

## Graphical abstract

**Figure:**
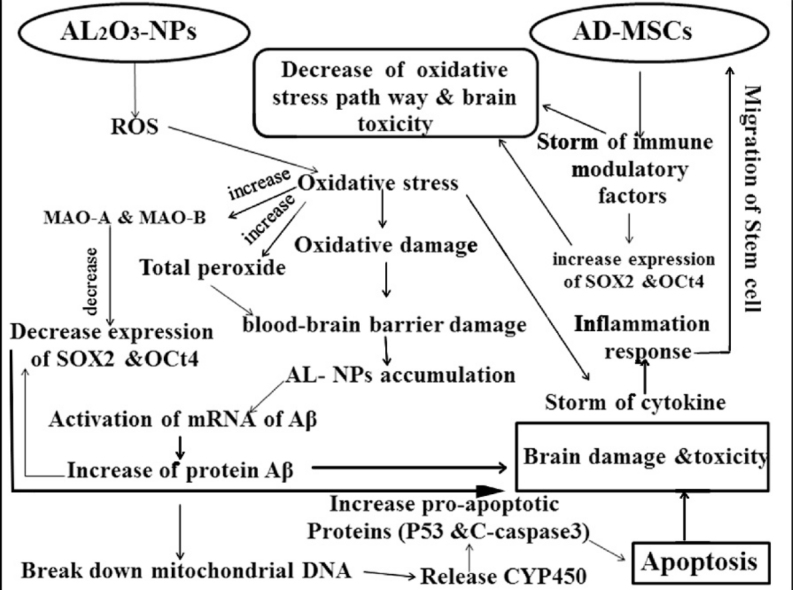

## 1 Introduction

Aluminum oxide nanoparticles (Al_2_O_3_-NPs) are used in many different clinical and industrial products; they have the potential to cause toxicity [1,2]. Such small particles can be taken up by cells and infiltrate the blood and lymph to induce injury [3]. An examination directed at the brains of different animals indicated that Al_2_O_3_-NPs induced oxidative stress and dysfunction of antioxidant enzyme-mediated defenses [4,5]. Furthermore, NPs caused severe damage to tissues, such as those of the liver, kidney, and the immune system [6], which stimulated the expression of pro-inflammatory cytokines and reactive oxygen species (ROS), as well as the mutation of DNA [7].

The use of nanotechnology for the treatment and control of biological systems generates potential toxicity in humans [8]. It is also noted that the toxicity is different in various body organs due to the accumulation of NPs in the tissues of such organs [9]. Moreover, the toxicity can even cross the blood–brain barrier (BBB) [10]. Al_2_O_3_-NPs in various rodents can cause neurotoxicity *via* cytotoxic, genotoxic, and inflammatory effects in the brain [11]. The main target by which Al_2_O_3_-NPs cause injury in the central nervous system is the brain. Al_2_O_3_-NPs deposited in different brain regions caused neurotoxicity and histopathological and ultrastructural damage in rats. Moreover, they decreased the viability of cells, caused mitochondrial dysfunction, inhibited cell cycle, and induced apoptosis in *in vitro* studies [12].

Al_2_O_3_ NPs have cytotoxic and genotoxic effects on CHO-K1 cells, as well as concentration-dependent inhibition of cell division in UMR106 cells [13,14] Furthermore, Al_2_O_3_ NPs exposed to pulmonary artery endothelial cells and human umbilical vein endothelial cells increased mRNA protein expression of a molecule, likely due to the generation of reactive oxygen species (ROS) and activation of redox-sensitive signaling pathways, which could be linked to cardiovascular health risks [15].

Mesenchymal stem cells (MSCs) are somewhat multipotent and undifferentiated stem cells. They can be isolated from numerous tissues, including the bone marrow, fat tissue, cord blood, and amniotic layer [16]. MSCs are attractive for use in clinical treatments, as they can be effectively isolated from almost all adult tissues [17] and have been shown to be safe and non-tumorigenic [[18], [19], [20]]. MSCs derived from the adipose tissue are desirable for clinical use as they are easy to extract and abundantly available [21]. AD-MSCs can differentiate into myocytes, hepatocytes, neural cells, osteocytes, chondrocytes, adipocytes, and epithelial cells of the lung, kidney, and skin [[22], [23], [24]]. In animal models, AD-MSCs have been utilized to alleviate hemorrhagic stroke [25] and spinal cord injury, reduce inflammation and neurodegeneration, enhance motor skills, and lower the immune system response [26]. The ability of AD-MSCs to repair tissues *via* their regenerative properties could restore damaged neural tissues, infected lung tissues, cystic fibrosis lung tissues [27], and wounds [28].

According to the immunomodulatory properties of MSCs, they may repair tissues and reduce oxidative stress *via* the expression of cytokines, chemokines, apoptosis inducers, and antitumor particles [29,30]. MSCs produce various trophic and developmental factors, influencing the neurogenesis, synaptogenesis, and astrocytosis factors [24]. Consequently, applications to protect tissues from the harmful effect of ROS have focused on stem cells [31]. In animal models, the effect of MSCs on organs, tissues, and the regulation of inflammation have been demonstrated [[32], [33], [34]]. Our current study illustrates the acute toxicity of Al_2_O_3_-NPs on the Aβ level, gene expressions of Sox2 and Oct4, and their effect on the brain. Also, the essential and vital pathways by which stem cells can treat damage or apoptosis induced by Al_2_O_3_-NPs in the hippocampal region of the rat brain.

## 2 Materials and methods

### 2.1 Materials

Aluminum oxide NPs were purchased from US Research Nanomaterials, USA (gamma, 99+%, average particle size 20 nm, hydrophilic, melting point determined *via* the high-temperature combustion method, Al_2_O_3_ SSA > 138 m^2^/g, morphology: nearly spherical, color: white). RPMI-1640 (with l-glutamine) growth medium, fetal bovine serum (FBS), and antibiotic mix were purchased from Gibco (Invitrogen, *ca.* USA). Collagenase Type II (Sigma-Aldrich, St. Louis, MO, USA) and mouse primary anti-CD105 and CD90.1 IgG and anti-CD45 antibodies were purchased from Thermo Fisher. Ultra-Tek polyvalent goat anti-mouse HRP was purchased from Sky Tek laboratories, Logan, Utah84323, USA. Polyacrylamide gel electrophoresis (SDS-PAGE) chemicals, ethylene glycol tetra acetic acid (EGTA), nitrocellulose membranes, protease inhibitors, mouse anti-p53, anti-cleaved caspase-3, anti-CYP2E1, and anti-Aβ, and goat anti-mouse IgG-HRP and goat anti-β actin IgG (USA), and monoamine oxidase A and B ELISA Kits were purchased from Sigma-Aldrich. SYBR green PCR Master Mix was purchased from USA.

### 2.2 Animal and experimental design

Adult female rats were kept at standard conditions (temperature at 23 ± 2 °C, lighting cycle 12 h light/dark; fed with standard chow and tap water) and were allowed to adapt to the laboratory housing conditions for 1 week prior to the start of the experiment.

The rats (*n* = 60) weighing 210 ± 50 g were divided into five groups: (I) a control group without any treatments; (II) a group receiving a daily oral dose of 9% NaCl; (III) a group receiving Al_2_O_3_-NPs (6 mg/kg body weight) [6] dissolved in 1 mL sodium chloride once daily for 20 days (The Al_2_O_3_-NPs solution was sonicated before each injection using a sonicator [35].); (IV) designated as (R), a group treated with Al_2_O_3_-NPs (6 mg/kg b.w.) dissolved in 9% NaCl solution once daily for 20 days, followed by a recovery period without treatment for an additional 20 days; and (V) designated as AD-MSCs-treated group, a group injected with 0.8 × 10^6^ AD-MSCs/0.5 mL phosphate-buffered saline (PBS) *via* the caudal vein after 20 days of oral treatment with Al_2_O_3_-NP. The rats were then sacrificed 5 days after MSC transplantation. The total experiment lasted 40 days.

#### 2.2.1 Ethical approval

Adult female rats were purchased and cared for by the Assiut University Joint Animal Breeding Unit according to the National Institutes of Health guidelines for the use of experimental animals. The committee of medical ethics of the Faculty of Medicine at Assiut University reviewed and approved the research procedures employed in this study (IRB no:17300503).

### 2.3 Characterization of aluminum oxide nanoparticles (Al_2_O_3_-NPs)

#### 2.3.1 X-ray diffraction (XRD) analysis

The crystal structures of powdered Al_2_O_3_-NPs were characterized by XRD by the Physics Department of Assiut University using a Philips X-ray diffractometer (Model PW 1710, Holland). The measurements were swapped from 2θ = 30° to 2θ = 80° using a copper X-ray tube operated at 40 kV and 40 mA, with a radiation wavelength of 1.5406 Å. The crystalline nature of the NPs was confirmed by the XRD pattern of Al_2_O_3_-NPs [36].

#### 2.3.2 Transmission electron microscopy (TEM) analysis

A drop of Al_2_O_3_-NPs (20 nm /100 μg/L) preparation for TEM to determine the size and shape of Al_2_O_3_-NPs as follows. First, the powder was dissolved in EtOH and then dispersed ultrasonically. Then, the particles were deposited on a carbon-coated copper grid and dried at room temperature. The micrographs of the samples were taken using a TEM at Assiut University’s Center of Electron Microscopy, Faculty of Science.

#### 2.3.3 Dynamic light scattering (DLS)

Mean particle size and polydispersity index (PDI) of the nanoparticles were measured at Assuit International Center of Nanoparticle (AICN), using a Zetasizer Nano ZS instrument (Malvern Instruments, Worcestershire, UK) equipped with a backscattered light detector operating at 173°. The Zeta-potential values were measured by laser Doppler anemometry using Malvern Zetasizer Nano series ZS. All samples were diluted in distilled water and measured at 25 °C in triplicates (equilibrium time of 120 s and 15 runs). The sample volume used for all measurements was kept constant.

### 2.4 Isolation of AD-MSCs from rats

The solid fat from the adipose tissue of adult male rats was cut into fine pieces and then washed with sterile PBS (Lonza, Swiss). Next, the pieces were then enzymatically digested by Collagenase Type II (0.25 % in PBS in 20 % FBS) for 45 min. During the digestion incubation at 37 °C, the Falcon tubes (50 mL) were shaken every 10 min, after which the collagenase activity was halted by the addition of FBS. The cell pellet containing the AD-MSCs was reconstituted in 12 mL of culture medium after centrifugation. The suspension was filtered by cell strainer (40 μm) and plated on 10-cm culture dishes. The cells were incubated at 37 °C, 5% CO_2_, for 2 weeks until the confluence reached nearly 80 %. AD-MSCs were subcultured for up to three passages [37].

### 2.5 Characterization of AD-MSC

#### 2.5.1 Immunocytochemistry

Paraformaldehyde (4%) was used to fix cells for 20 min at room temperature. Cells were washed in PBS 3 times for 5 min each. The cells were permeabilized using fresh 0.2 % Triton X-100 in PBS for 5 min and then washed three times with PBS for 5 min each [38]. Secondary anti-polyvalent stain was also used as per the manufacturer’s protocols. First, nonspecific background staining was reduced by incubating the slides in blocking buffer for 10 min and then washing two times with PBS. Next, the slides were incubated in primary antibodies against CD105, CD90 (2:100), and CD45 (1:100) for 1 h at room temperature and then washed four times with PBS buffer for 5 min each. Ultra-Tek anti-polyvalent stain was applied and incubated for 10 min at room temperature and then washed four times. DAB chromogen was added to the DAB substrate mix, and the slides were incubated in the solution for 5 min and then immediately counterstained and coverslipped.

#### 2.5.2 Flow cytometry analysis for the characterization of AD-MSCs

Flow cytometry was performed by the Assiut University Faculty of Medicine. AD-MSCs weretrypsinized (1% trypsin-EDTA, Sigma-Aldrich) after the third passage and thencentrifuged. The cell pellets (1 μg/10^6^ cell) were suspended in 1% FBS/PBS for 30 min on ice and then incubated in fluorescein isothiocyanate (FITC)-conjugated anti-rat CD27 and CD105, or CD45 and CD34 monoclonal antibodies (BD Pharmingen, San Diego, CA, USA)Data were analyzed using the FCS Express 7 software [39,40].

### 2.6 Western blot analysis

Brain samples were homogenized in RIPA Lysis Buffer (1% Nonidet-P40, 1% Triton X-100, 0.5 % Na deoxycholate, 150 mM NaCl, 1 mM PMSF, 5 mM EDTA, 10 mM EGTA, 50 mM Tris−HCl, and 1% leupeptin/pepstatin protease inhibitor cocktail). The protein concentration was estimated. SDS-PAGE (10 %) was utilized to resolve protein aliquots, which were transferred onto a nitrocellulose membrane. The membranes were blocked with 5% skim milk in TBS containing 0.05 % Tween 20 and then incubated with primary antibodies overnight at 4 °C. Subsequently, the membranes were incubated with HRP-conjugated secondary antibodies in the blocking solution for 1 h at 24 °C. A Chemiluminescent Substrate Kit was used to visualize immunodetected bands. The anti-actin goat polyclonal antibody and Rb anti-goat HRP-conjugated antibody were used for the confirmation of equal loading. The data are expressed as mean ± SE from at least three separate experiments; the optical density of the bands was estimated as uncalibrated optical density using the ImageJ software [41].

### 2.7 Immunohistochemistry study

Paraffin-embedded tissues were deparaffinized in xylene, rehydrated in a series of ethanol solutions (100 % to 70 %), and submerged in water. Antigens were retrieved by boiling the slides in 1 mM EDTA, developing sections in 3% H_2_O_2_ for 10 min, washing with wash buffer (1X PBS) for 5 min, and then blocking each section at room temperature with 100–400 μL blocking solution for 1 h. The blocking solution was removed, and cleaved caspase 3 primary antibody was added (1:10). The antibody solution was removed, and the sections were washed for 10 min with wash buffer. Secondary antibodies were applied (1: 5000) to each incubated portion for 30 min and then removed. The sections were washed and then stained for 2–3 min with 3, 3′-diaminobenzidine (DAB) and counterstained with hematoxylin for 2–5 min. The reaction was immediately quenched in distilled water. A light microscope was used to visualize the stained sections [42].

### 2.8 Quantitative real-time PCR (qRT-PCR)

RNA was extracted from brain samples using the RNA Simple Mini Kit (Invitrogen). Reverse transcription was performed using the SMART_PCR cDNA synthesis kit (Clontech Inc., Palo Alto, CA). Quantitative real-time polymerase chain reaction (qRT-PCR) was performed in duplicate in 25-μL reaction mixtures containing 1-μL cDNA template, SYBR Green PCR Master Mix, and 10 pmol of each primer Master Mix. The sequences of primers are as follows: *Sox2* forward, 5′-AAGGGTTCTTGCTGGGTTTT-3′ and reverse, 5′-ACGAAAATGGTCTTGCCAG-3′, *Oct4* forward, 5′-TGTTCCTGTCACTGCTCTGG-3′ and reverse 5′−CCCCTGTTTGTGCTTTCAAT-3′ [43], and *GAPDH* forward, 5′-AACTTTGGCATTGTGGAAGG-3′ and reverse, 5′-GTCTTCTGGGTGGCAGTGAT-3′. Reactions were performed in an I Cycler iQ (Bio-Rad). The level of each cDNA amplicon was standardized to that of *GAPDH* mRNA in the equivalent sample. The data are expressed as mean ± SE from at least three separate experiments

### 2.9 Chromosome detection (SRY gene) by PCR

Three days after the AD-MSCs transplantation, PCR was employed to identify rat Y-chromosome-specific SRY genes from the brains of female rats. DNA was extracted from the brain using the QIAamp Tissue Kit (Qiagen, Valencia, CA, USA) as per the manufacturer’s protocols. First-strand DNA was elongated for 1 h at 42 °C. Then, DNA strand amplification was performed *via* 35 thermal cycles consisting of 95 °C denaturing for 30 s, 60 °C annealing for 45 s, and 72 °C extension for 2 min. PCR products were then separated by 1% agarose gel electrophoresis, stained with ethidium bromide, and visualized under UV transillumination. The sequences for the SRY gene were 5′-CATGAACGCATTCATCGTGTGGTC-3′and5′CTGCGGGAAGCAAACTGCAATTCTT-3′ [44].

### 2.10 Determination of monoamine oxidase (MAO)-A and B levels and total peroxide

The activity of MAO-A and B was determined using ELISA Kit according to the manufacturer’s protocols (USA). In a microplate reader (Synergy HT; BioTek, USA), absorbance was measured using excitation within the 530/25-nm range and 590/20-nm emission detection [45]. The total peroxide content was assayed with xylenol orange as follows: first, 30 μL of tissue homogenate was incubated in 1 mL of 2.4 mg of FeSO_4_. Then, 2.5 mL of distilled water, 65 μL of H_2_SO_4_, 22.5 mL of absolute ethanol containing 20 mg of butylhydroxytoluene, and 2 mg of xylenol orange was prepared at room temperature; tissue homogenate was reacted for 30 min [46].

### 2.11 Histological examination and histopathology scores

For histological and histopathological examinations, tiny pieces of brain tissue were fixed in 10 % neutral formalin (pH 7.2). Paraffin sections with a thickness of 5 μm were prepared and then stained with hematoxylin and eosin. Five parameters of brain histopathology (cytoplasmic vacuolization, region of degeneration, nuclear condensation, nuclear fragmentation, and inflammation) were scored for brain injury and histopathology [47] using the ImageJ software.

### 2.12 Statistical analysis

The results from all the quantitative data are indicative of at least three independent determinations. Values are expressed as mean ± SE. Student’s *t*-test was conducted to compare the parameters between two groups. Statistical analyses were conducted using analysis of variance, and the difference was deemed significant at *p* <  0.001. The outcomes of the NaCl group were correspondingly similar to those of the control group; thus, only the results of the control group were shown.

## 3 Results

### 3.1 X-ray diffraction (XRD) of Al_2_O_3_-NPs

**Fig. 1:**
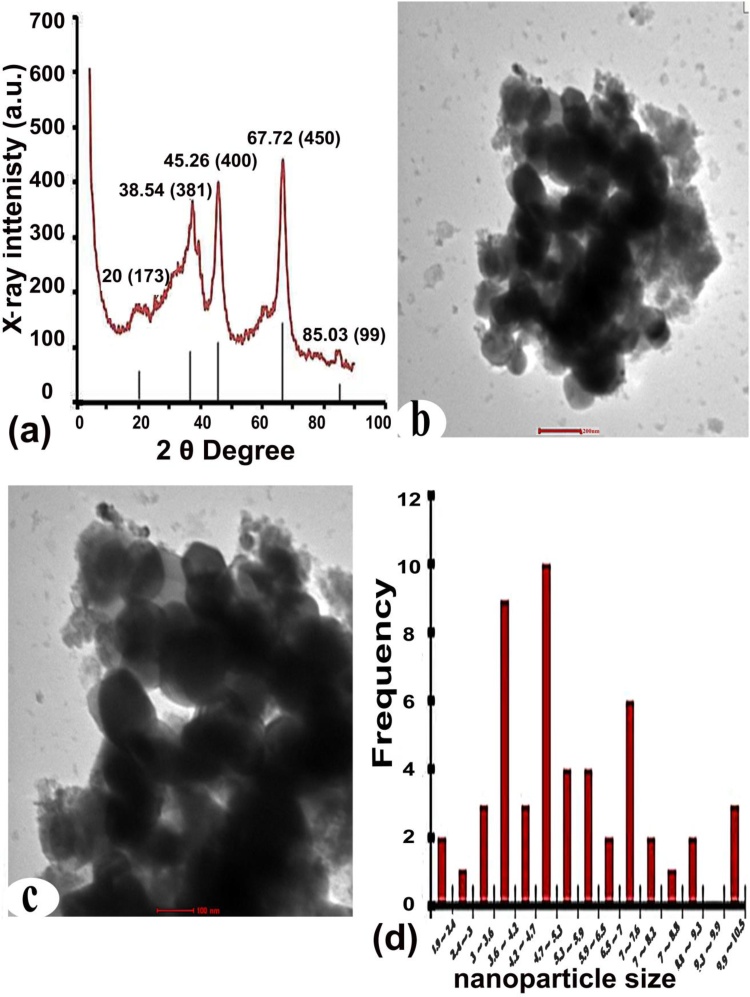
**(a)** X-ray diffraction pattern analysis indicating the uniform size of aluminum oxide nanoparticles (gamma, 99+%, 20 nm, hydrophilic). **(b–c)** Transmission electron microscopy analysis aqueous dried 20 nm/100 μg/L Al_2_O_3_-NPs (scale bar: 200, 100 nm) and a histogram of size distribution **(d)**.

Five Bragg reflections corresponding to 20°(173), 38.54°(381), 45.26°(400), 67.72°(450), and 85.03(99) were observed on sets of lattice planes, respectively. These were indexed according to the facets of the face-centered cubic (fcc) crystal structure of Al_2_O_3_-NPs (Research Nanomaterials USA, stock US3023) **(**Fig. 1a**)**.

### 3.2 Transmission electron microscopy (TEM)

The diameters and frequency distributions of Al_2_O_3_-NPs are presented in Fig. 1b-c. The average particle size and SD were 38.31 ± 2.45 nm (*n* = 60) for 20 nm of Al_2_O_3_-NPs.

### 3.3 Dynamic light scattering (DLS)

The particle size in terms of intensity and numbers are shown in Table 1. Since DLS calculates the hydrodynamic diameter of the particles, the particle size measured by DLS was bigger than that measured by TEM micrographs.

**Table 1:** Dynamic light scattering measurements:

PDI	Average particle size by number (n.m)	Average particle size by intensity (n.m)	Formulation
0.239 ± 0.029	1577 ± 120.2	2257 ± 188.3	1

### 3.4 Identification and characterization of AD-MSCs

**Fig. 2:**
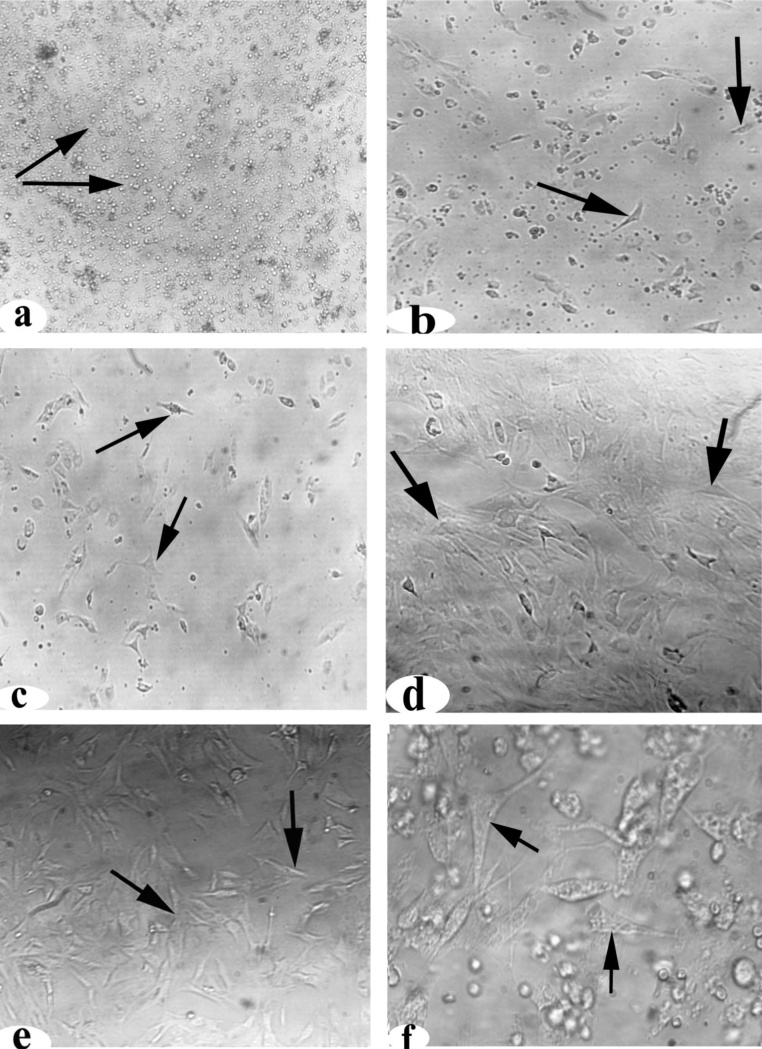
Detection of AD-MSCs *via* phase-contrast microscopy on different days and passages. **(a)** Day 0, **(b)** day 1, **(c)** passage (P)1, **(d)** P2, **(e)** P3 (200X), and **(f)** P3 (400×). AD-MSCs were characterized by expansion and morphology at different passages. The arrows indicate fibroblastic appearance.

Immediately after isolation at culture day 0, AD-MSCs appeared circular and were in suspension **(**Fig. 2a**).** After 1 day of differentiation, the cells started to attach in a thin spindle shape **(**Fig. 2b**).** AD-MSCs were differentiated in different passages: in passage one (P1), some of the cells appeared spindle-shaped **(**Fig. 2c**)**; in passage two (P2), the cells formed small colonies **(**Fig. 2d**)**; and in passage three (P3), the cells had fibroblastic appearances (Fig. 2e, 200×, and Fig. 2f, 400×).

**Fig. 3:**
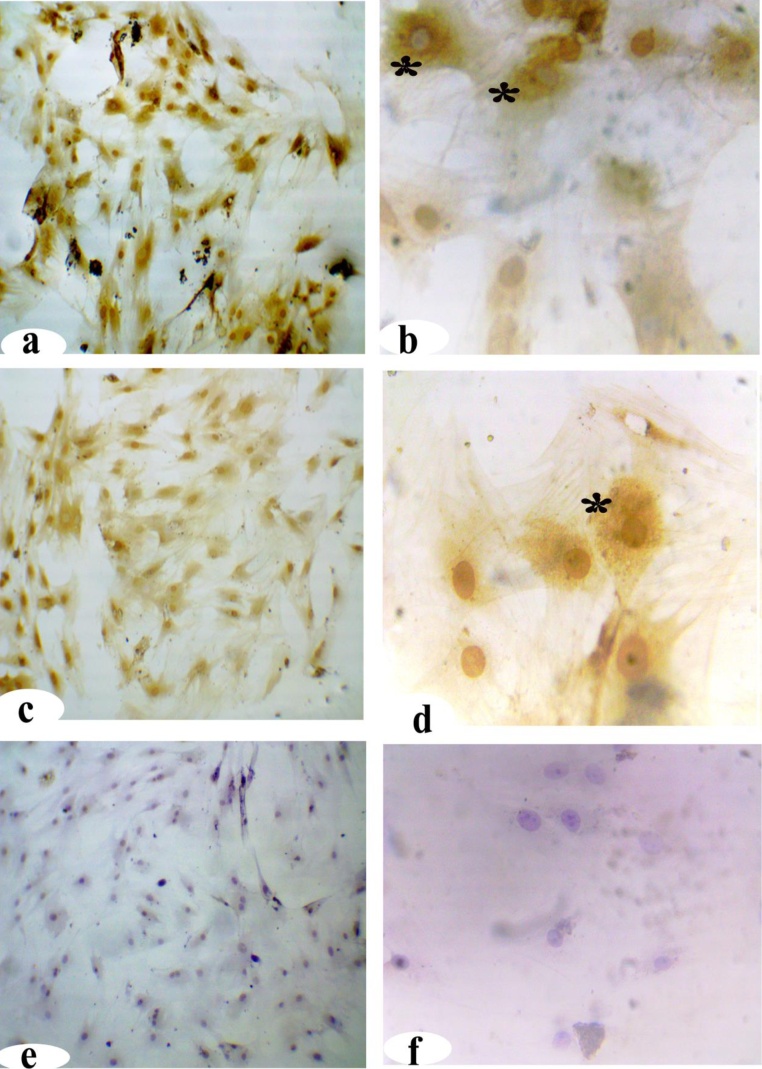
Expression of AD-MSC markers by immunocytochemistry. **(a–b)** CD105 and **(c–d)** CD90 were used as positive markers (*), and **(e–f)** CD45 was used as a negative marker (200× and 400×).

The immunocytochemistry of AD-MSCs at P3 showed a strong positive detection of CD105 (Fig. 3**a–b**) and CD90 (Fig. 3**c–d,** 200× and 400×). The brown dots (positive) localized to the AD-MSC nuclei and the cytoplasm in contrast to CD45, which was not detected there (Fig. 3**e–f,** 200× and 400×).

**Fig. 4:**
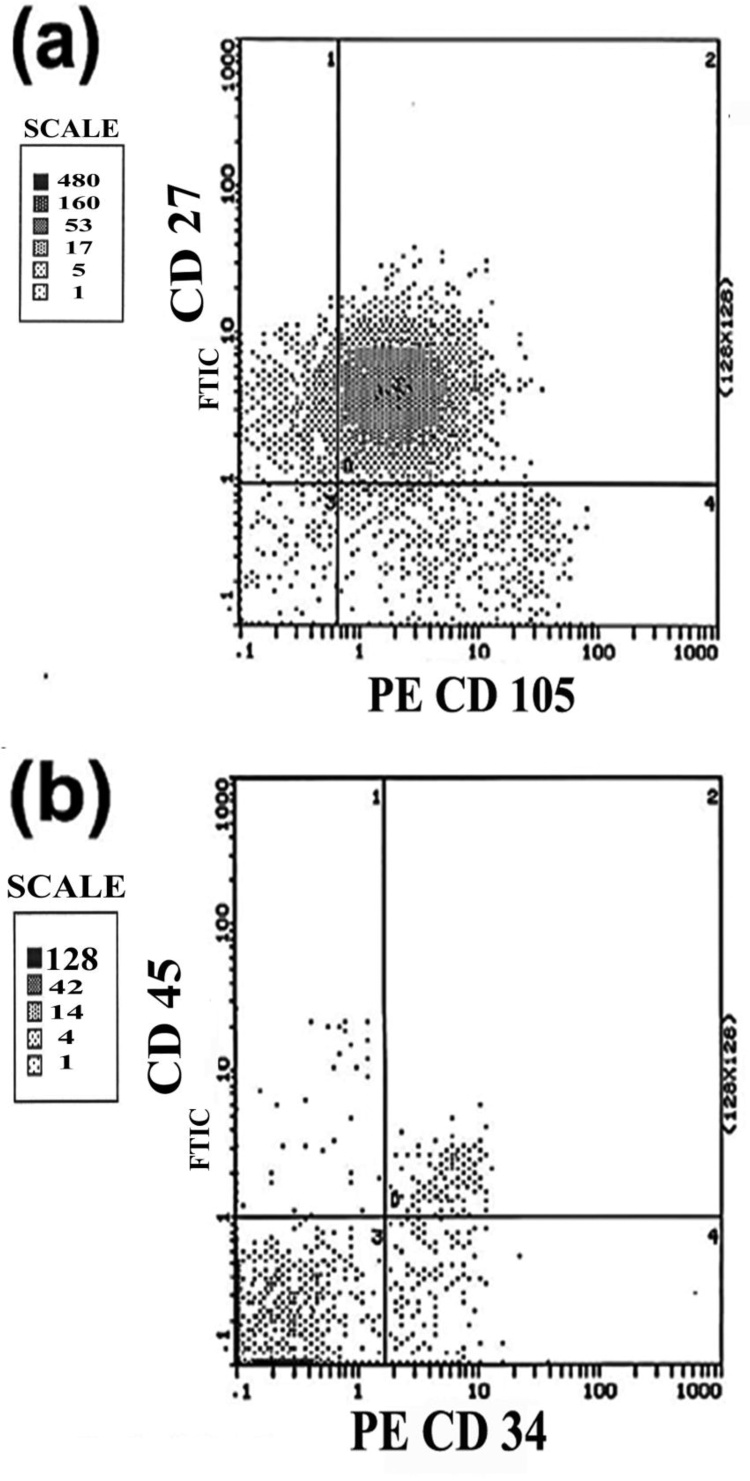
Flow cytometric analyses of AD-MSCs. **(a)** Cells were positive for the expression of CD27 and CD105, and **(b)** cells were negative for the expression of CD45 and CD34.

The identity of AD-MSCs was confirmed by dual flow cytometry analysis at passage 3. The cell surface of at least 98 % of AD-MSCs demonstrated the expression of stem cell markers, especially CD27 and CD105 (Fig. 4a). Contrarily, less than 1% of the cell populations showed CD45 or CD34 expression (Fig. 4b).

### 3.5 Determination of apoptotic proteins, P450 (CYP2E1), and Aβ

**Fig. 5:**
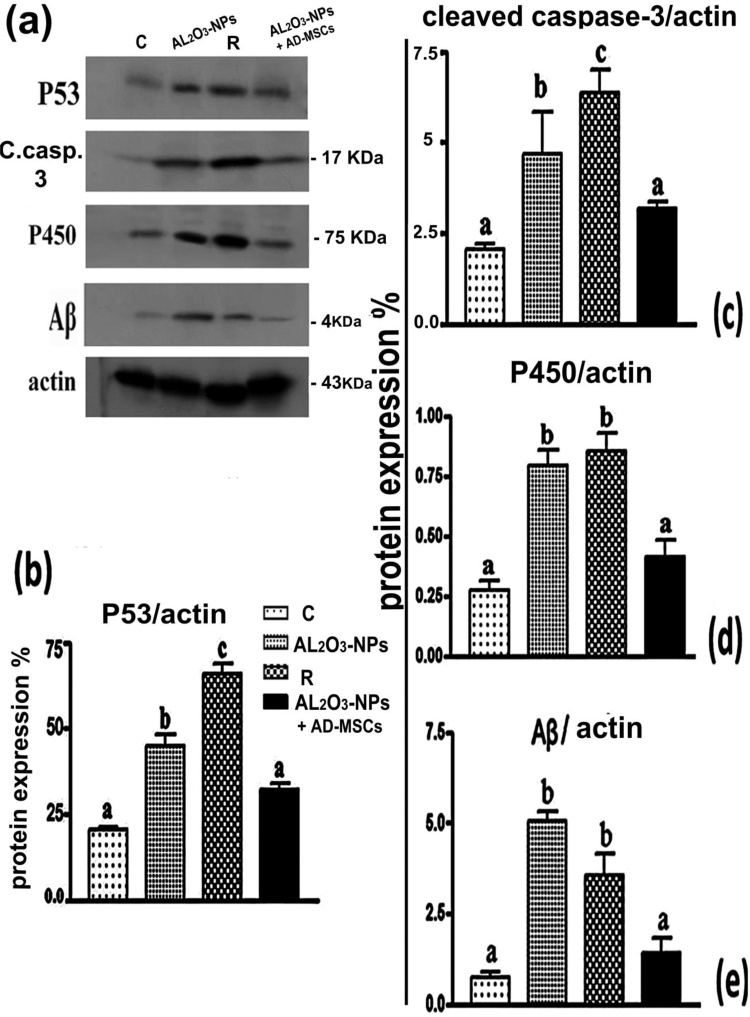
Immunoblot and quantified densitometric analysis conducted to assess the effects of AL_2_O_3_-NP exposure and various treatments on the changes in the protein levels of P53, cleaved caspase-3, CYP1A1 (P450), and Aβ. The corresponding antibodies were used to assess the changes in the protein levels. Equal protein loading was confirmed by reprobing with anti-actin antibodies as a protein loading control. **(a–e)** Values with different letters indicate significant differences (*p* <  0.001).

Western blot detection revealed changes in protein levels in the brains of rats treated with Al_2_O_3_-NPs (6 mg/kg) for 20 days. The Al_2_O_3_-NPs and recovery rats exhibited significantly increased levels of p53, cleaved caspase-3, P450, and Aβ (117.7 %, 127.5 %, 190.3 %, and 589.4 % and 219.4 %, 208.1 %, 212.2 %, and 381.9 %, respectively) compared with the control. Also, co-treatment of Al_2_O_3_-NPs and AD-MSCs significantly decreased protein levels close to the levels of the control group (29.0 %, 32.0 %, 47.7 %, and 72.3 % compared with the Al_2_O_3_-NPs-treated rats) (Fig. 5**a–e**).

### 3.6 Immunohistochemistry (IHC) detection of cleaved caspase-3

**Fig. 6:**
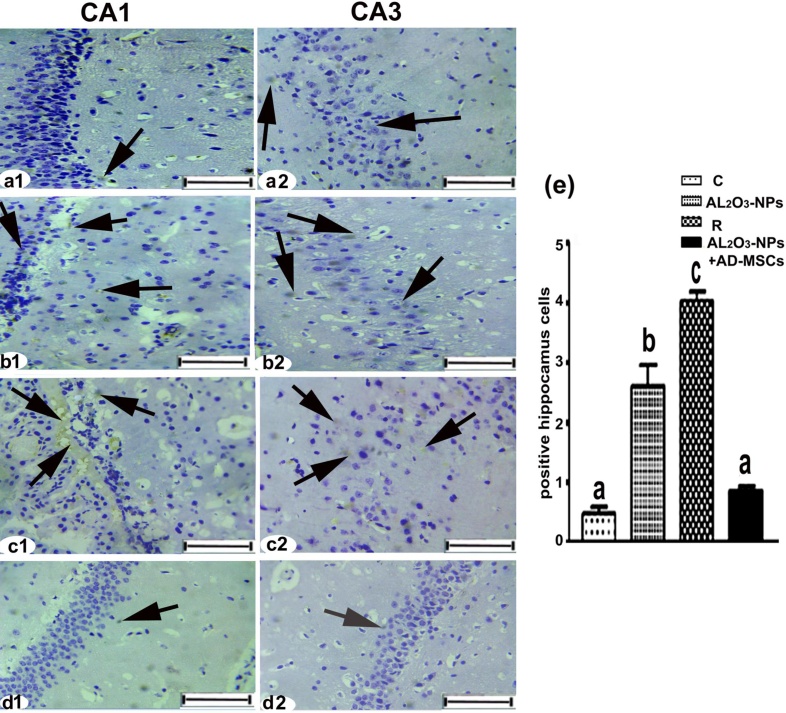
**(a1–a2)** Immunohistochemical staining of cleaved caspase-3 in the CA1 region and the CA3 region of the brain hippocampal cells. **(b1–b2)** The AL_2_O_3_-NP-treated group showed brown immunoreactive staining of neurocytes (arrows). **(c1–c2)** The R group showed severe positive immunoreactive staining of neurocytes (arrows). **(d1–d2)** The AL_2_O_3_-NPs + AD-MSCs group showed negative or moderate immunoreactivity of neurocytes (brown patches, arrows).Scale bar: 50 μm. **(e)** Statistically, the values in the column with unlike superscript letters were significantly different (*p* < 0.001).

Immunoperoxidase DAP staining was negative for immunoreactivity against cleaved caspase-3 in the control group in neurocytes and pyramidal cells in the vast majority of the hippocampus (Fig. 6**a1–a2**).Immune reaction against cleaved caspase-3 demonstrated a sharp increase and large homogeneous brown patches in the neurocytes of the hippocampal region in Al_2_O_3_-NPs-treated group (Fig. 6**b1–b2, e**) and the recovery group (Fig. 6**c1–c2, e**). Densitometry calculation revealed that the levels of cleaved caspase-3 increased by 457.1 % and 762.0 %, respectively, compared with the control group. In the Al_2_O_3_-NP + AD-MSCs group (Fig. 6**d1–d2, e**), no brown patches or only a few were observed in the hippocampal region, confirming the reduction of cleaved caspase-3 (67.3 % compared with the Al_2_O_3_-NP-treated group).

### 3.7 Enhanced expression of *Sox2* and *Oct4* by AD-MSCs

**Fig. 7:**
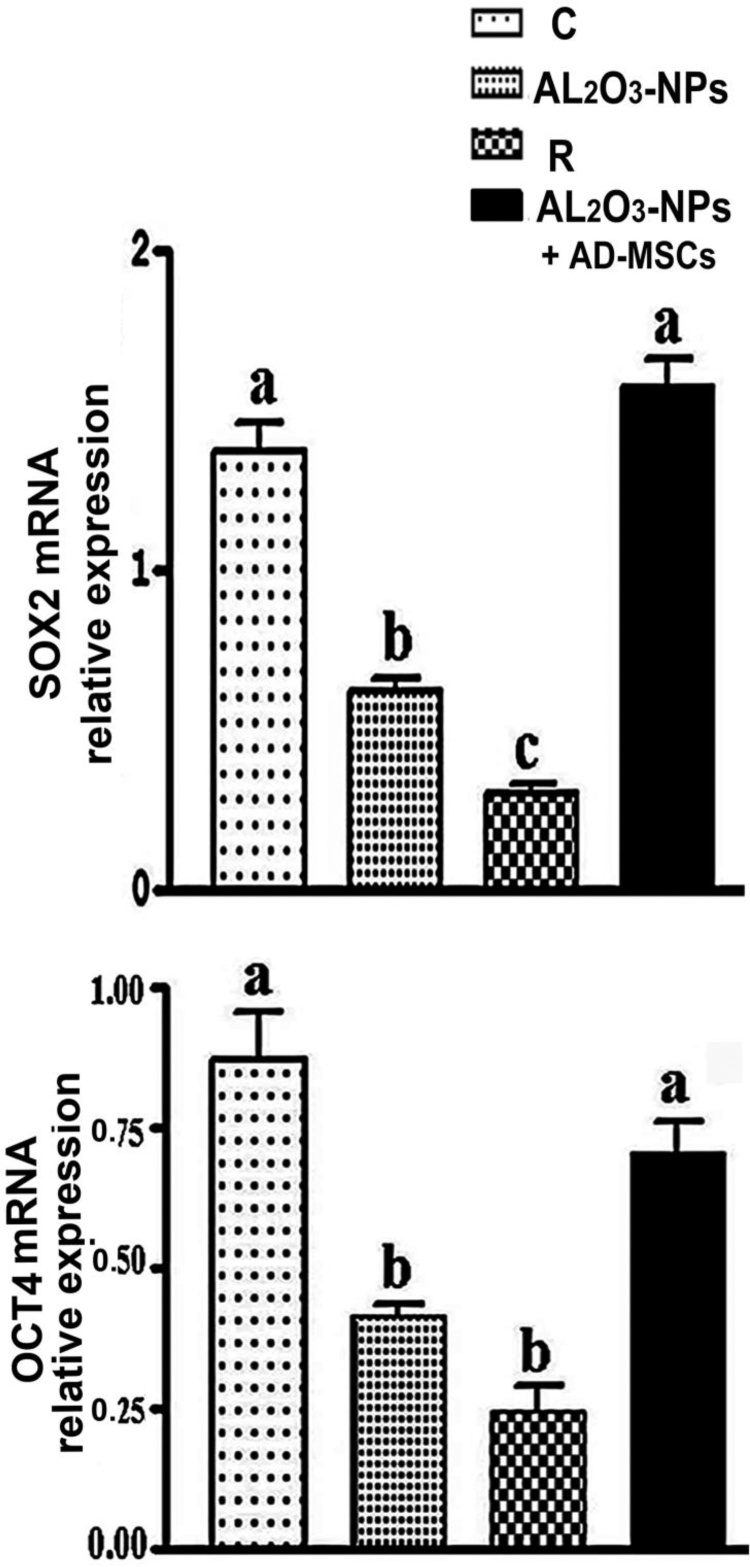
**(a–b)** qRT-PCR and expression analysis of *Sox2* and *Oct4* in different treatments. Results and values were normalized to *GAPDH* mRNA levels.

The levels of *Sox2* and *Oct4* mRNA were quantified in the brain tissues by qRT-PCR. These levels were significantly increased by 1.0- and 0.3-fold, respectively, in the Al_2_O_3_-NPs + AD-MSC rats *versus* the Al_2_O_3_-NPs-treated group. Contrarily, the levels of *Sox2* and *Oct4* mRNA were significantly decreased in the Al_2_O_3_-NPs and recovery groups (0.8- and 0.5-fold and 1.07- and 0.6-fold, respectively) compared with the control group. This observation confirms the enhanced expression of *Sox2* and *Oct4* by AD-MSCs and the immune storm stimulated by AD-MSCs against the toxicity of Al_2_O_3_-NPs (Fig. 7**a–b**).

### 3.8 Y-chromosome-specific gene detection in the brain tissue of female rats

**Fig. 8:**
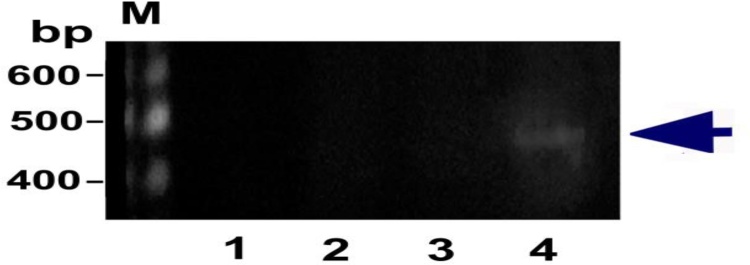
Agarose gel electrophoresis for PCR products of the *SRY* gene in the brain tissues of different groups. *SRY*-positive DNA marker in lane M; groups 1, 2, and 3 were negative for *SRY*, and group 4 was positive for the *SRY* gene in lane 4 after the female rats received AD-MSCs from male rats (*GADPH* served as an internal reference gene).

The PCR products of the *SRY* gene in the brain tissues of all groups are presented in Fig. 8. The *SRY* gene of male rats could be detected in the brain homogenates of female rats that received AD-MSCs transplantation. Lane 4 shows that AD-MSCs can migrate to the site of injury in the brain. However, no *SRY* gene products were detected in lanes 1, 2, and 3.

### 3.9 Measurements of the total peroxide levels and MAO-A and MAO-B activities

**Fig. 9:**
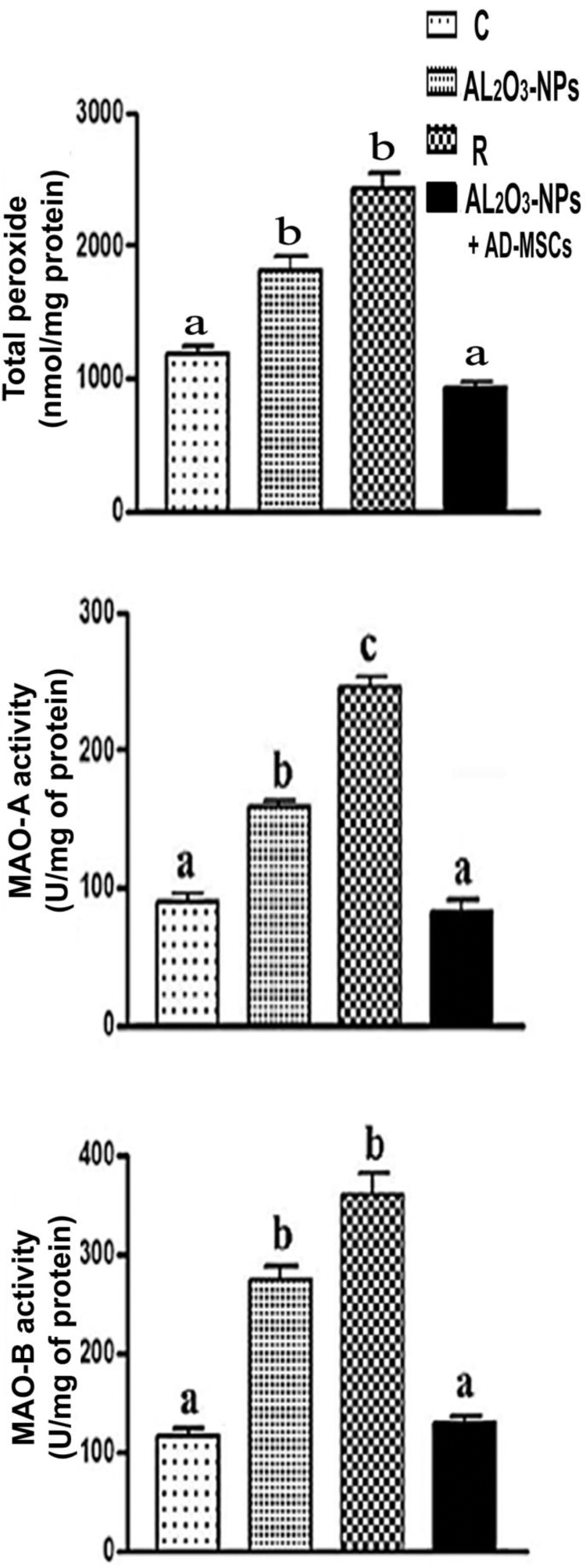
The total peroxide levels and MAO-A and MAO-B activities in the brain homogenates of the control and treated rats. Values indicated by unlike letters were significantly different (*p* < 0.001).

The level of the brain total peroxide and MAO-A and MAO-B activities in female rats administered Al_2_O_3_-NPs and recovery treatments were significantly upregulated (52.8 %, 43.77 %, and 136.2 %, and 105.1 %, 175.3 %, and 211.1 %, respectively) compared with the control. However, the Al_2_O_3_-NPs + AD-MSCs-treated group exhibited a significant downregulation of protein levels and activities by 49.1 %, 48.0 %, and 52.4 %, respectively, compared with the Al_2_O_3_-NPs-treated group (Fig. 9).

### 3.10 Light microscopic examination

**Fig. 10:**
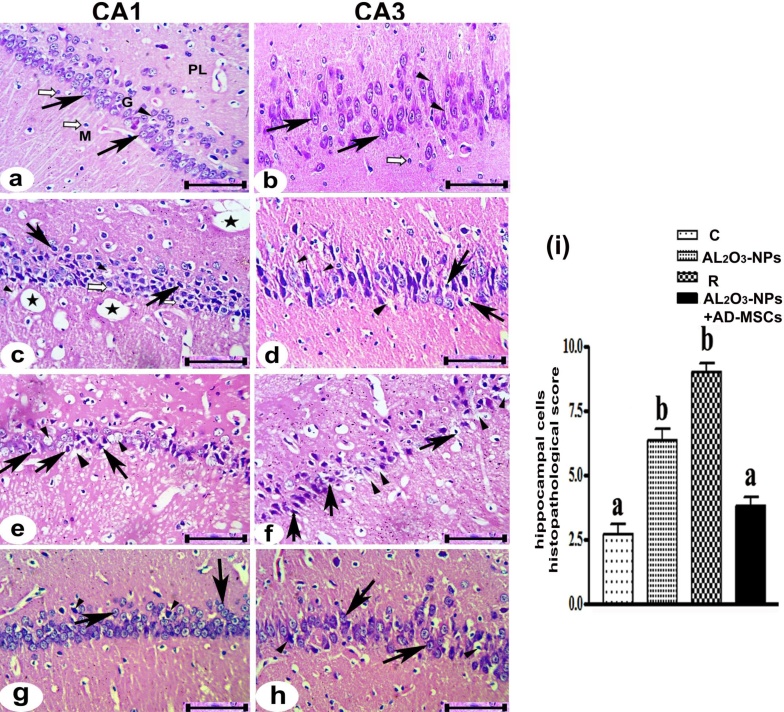
Photomicrographs in the CA1 region **(a, c, e,** and **g)** and the CA3 region **(b, d, f,** and **h)** of the hippocampus proper. **(a)** The control group, showing polymorphic layer (PL), granular layer (G), molecular layer (M), small pyramidal cells (↑), neural processes (▲), and glial cells (white arrow). **(b)** The control group, showing large pyramidal cells (↑), large neural processes (▲), and glial cells (white arrow). **(c–d)**The Al_2_O_3_-NPs-treated group showing degenerated and shrunken pyramidal cells surrounded by spaces (↑), vacuolated cytoplasm (white arrow), rarefied areas (▲), and dilated blood vessels (asterisk). **(e–f)** The recovery group showing degenerated pyramidal cells (↑) and many rarefied areas (▲). **(g–h)** The Al_2_O_3_-NP + AD-MSCs group showing pyramidal cells with vesicular nuclei and prominent processes (↑) and a few shrunken pyramidal cells with condensed nuclei (▲). (H&E stain, scale bar =50 μm). **(i)** The histopathology score of the liver was measured as defined in the methods section. The results are presented as the mean standard error of at least three separate experiments. The letters of the columns are significantly different (*p* < 0.001).

The control group exhibited proper hippocampal histochemical structure made up of a polymorphic layer, a granular layer, and a molecular layer. The granular layer mainly consists of neurons with small processes in the CA1 region, which are called small pyramidal cells (Fig. 10a), and large processes in the CA3 region, which are called large pyramidal cells (Fig. 10b). Between the pyramidal cells and in the molecular layer, there are glial cells. The hippocampus exhibited several dystrophic changes after treatment with Al_2_O_3_-NPs, characterized by small cell degeneration and shrinkage which surrounded by empty space, and large pyramidal cells with pycnotic and condensed nuclei in the CA1 **(**Fig. 10c**)** and CA3 **(**Fig. 10d**)** regions, respectively. In some pyramidal cells, the cytoplasm was also highly vacuolated. Between pyramidal cells and the dilatation of blood vessels in the polymorphic and molecular layers, several rarefied areas were observed. The recovery group demonstrated persistence of the harmful effects of Al_2_O_3_-NPs by a marked reduction in the granular layer thickness and a decline in the number of pyramidal cells in the granular layer in the CA1 (Fig. 10e) and CA3 (Fig. 10f) regions.

Multiple pyramidal degenerated cells were observed upon treatment with Al_2_O_3_-NP. Compared with those in the Al_2_O_3_-NPs-treated group, there was an increase in the number of rarefied areas. Stem cell treatment (Fig. 10**g–h**) exhibited a marked improvement in pyramidal cells, especially in small pyramidal cells. With prominent processes similar to those in the control group, the vast majority of the pyramidal cells demonstrated vesicular and rounded nuclei. However, a few pyramidal cells (small and large) were still reduced with condensed nuclei and dense cytoplasm. The histopathological score of the hippocampal cells was evaluated using Heijnen’s method; the highest scores were obtained in the Al_2_O_3_-NPs-treated and R groups. Experimental groups treated with AD-MSCs exhibited a substantial reduction in score relative to the other groups (Fig. 10I).

## 4 Discussion

The present study found that AD-MSCs can rescue the hippocampus against the deleterious effects of Al_2_O_3_-NPs. These data are in agreement with the different reports on the role of MSCs having antioxidant, immunomodulatory, and anti-inflammatory effects [48]. It has been demonstrated that AD-MSCs promoted neuroectodermal differentiation and repair and reduced apoptotic protein levels [49,50]. MSCs also led to the downregulation of both caspase-3 mRNA expression and Bax protein expression after injection following treatment with cisplatin [24,51]. Wen et al. [52] confirmed that bone marrow MSCs also have defensive efficacy and reduced oxidation (malondialdehyde levels) and cytochrome c, eliminating neural cell damage as a result of oxidative stress reduction. These cells secreted different factors, such as nerve growth factors and brain-derived neurotrophic factor, which are major tools in the treatment of neurological damage.

The current investigation found that AL_2_O_3_-NPs treatment and recovery led to the stimulation of ROS and oxidative stress. The high deposition of Aβ protein in the hippocampal region of the brain is an indicator of ROS. The high levels of Aβ protein induce apoptosis through the activation of proapoptotic pathways *via* cleaved caspase-3, P53, and P450. Also, the co-administration of AD-MSCs decreased apoptotic cell death by decreasing the levels of these proteins in the hippocampal region of the brain. Bodart-Santos et al. [53] used MSCs of human Wharton’s jelly to protect the hippocampal neurons in an Aβ-exposed cell culture. These cells could prevent synapse damage caused by amyloid-β and oxidative stress. In rats, a significant change was observed in the mRNA expression of Aβ, CYP450 enzyme *CYP 1A2*, and oxidative stress markers following oral exposure to a high dose of nano-copper [54]. Aβ accumulation induced mutation in mitochondrial DNA, which led to mitochondrial dysfunction [8,55] and initiation of lipid peroxidation [43].

Cell apoptosis was induced *via* Aβ deposition in the brain, leading to cleavage of caspase-3, which is capable of cleaving genomic DNA during apoptosis [34]. Liu et al. [12] revealed that nano-Al_2_O_3_ reached the brain *via* the olfactory nerve pathway, resulting in a severe decrease in the expression of Bcl-2 and Mdm2 and stimulating P53 and P21 expression. These observations are supported by reports that nano-alumina and NPs can alter the ability of the mitochondrial membrane to perform its function. Also, they can trigger oxidative stress, disrupt integrity, and decrease protein secretion [56]. Al-NPs can also alter the brain membrane by reducing the integrity of lipoproteins, and partial BBR damage caused by Al accumulation causes damage to both the hippocampi of rats *in vivo* [5] and human hepatocellular carcinoma cells [57] and liver and kidney cells [58,59].

Our results indicate an increase in brain *Sox2* and *Oct4* expression levels after the administration of AD-MSCs compared with the Al_2_O_3_-NPs-treated group nearly like control. Accordingly, other data indicated that SOX2 overexpression, which enhances OCT4 expression through genetic modification, improved brain neuronal differentiation. Sox2 and its family members activate signaling pathway molecules and pluripotent transcription factors that directly or indirectly influence neuron protection as well as control its expression to treat brain tumors in humans and mice [60].

Alshatwi et al. [61] were observed ANP toxicity in human mesenchymal stem cells (hMSCs) may be induced by an increase in oxidative stress after only 24 h of exposure and there were dose-dependent effects. In response to ANPs, the expression levels of oxidative stress-responsive enzymes CYP1A were up-regulated, and the antioxidant enzyme SOD expression was found to be significantly reduced. That confirms our result, after a 5-day of AD-MSCs injection *via* caudal vein they reduce the toxicity of AL_2_O_3_ NP due to its immunomodulatory response.

AD-MSCs also release different trophic factors that attenuate neuroinflammation, promote angiogenesis and neurogenesis, and reduce apoptosis. MSCs are induced to secrete anti-inflammatory factors *via* pro-inflammatory signals, such as lipopolysaccharide, tumor necrosis factor-α, and nitric oxide [62]. AD-MSCs are largely capable of treating hidden damage *via* a variety of anti-inflammatory cytokines and chemokines [26]. Most MSCs do not cross the BBB to the site of injury but instead modify periphery immunologic factors [63,64]. For example, prostaglandin E2 serves as a potent immunomodulatory factor that is constitutively expressed *via* the COX-2 anti-inflammatory pathway [65]. In the injured gastric mucosa in rats, BM-MSCs and AD-MSCs have been identified by the *SRY* gene [66], which is in agreement with our results that some AD-MSCs will move to the site of brain injury after BBR damage.

Another explanation was observed the role of AD-MSCs to reduce the effect of AL_2_O_3_-NP may be due to the reduction of microglia activation. Microglia are the brain’s resident immune cells and play an important role in neuroinflammation and, as a consequence of injury, undergo phenotypic transformation and activation [67]. Microglia activation is associated with chronic neuronal inflammation following brain injury. Activated microglia secrete IL-1α and TNF-α after injury, which stimulate neurotoxic reactive astrocytes, restricting neuron survival, outgrowth, and synaptogenesis [68]. M1 phenotype microglia release pro-inflammatory cytokines and oxidative mediators, whereas M2 phenotype microglia release anti-inflammatory cytokines and neurotrophic factors [69]. Ruppert et al. [70] found that AD-MSCs effectively reduced the number of M1 microglia 3 days after injury. However, the percentage of M2 microglia was increased 14 days after injury, indicating an anti-inflammatory change.

Recently, chronic exposure to ethanol in rats was demonstrated to increase MAO-A and MAO-B protein activities that stimulated the proapoptotic cascade in renal epithelial cells. These cells were the key source of ROS, H_2_O_2_ generation, and mitochondrial cytochrome c release [71]. Total peroxide is also a possible destructive ROS marker and has been shown to be generated in pathological conditions at high concentrations [72]. Our findings are in line with those of previous researchers in terms of the elevation of these proteins and their activities due to Al_2_O_3_-NP exposure. However, after AD-MSCs co-administration, a return of total peroxide levels & MAO-A & MAO-B activities nearly to the normal state was observed. All of our Al_2_O_3_-NP exposure outcomes were supported by histopathological changes in the hippocampal neurons. Other findings in various tissues also supported our observations, whereas acute doses of Al-NPs and AlCl_3_ have been demonstrated to cause histopathological alterations in the liver, brain, and kidney [58,73]. The restoration of the cerebral cortex structure and hippocampal cells in aluminum-administered rats to the normal state has also been demonstrated due to the endogenous nerve growth factors and immunomodulatory influenced by BM-MSCs [23,74].

## 5 Conclusions

Our findings indicate that exposure to and recovery from Al_2_O_3_-NPs induced toxicity in the hippocampal region of the brain. Al_2_O_3_-NPs were responsible for the induction of apoptosis *via* two pathways: stimulation of oxidative stress and increase in Aβ protein levels. The high level of Aβ induces apoptosis directly by activating proapoptotic pathways *via* cleaved caspase-3, P53, and P450 and indirectly by increasing the levels of total peroxide and the activities of MAO-A and MAO-B as well as decreasing the expression of Sox2 and Oct4 proteins. The co-administration of AD-MSCs reduced toxicity induced by aluminum oxide nanoparticles and restored the expression of the above proteins to their normal state by regulating the levels of Sox2 and Oct4.

## Author contributions

**Mona M. Atia**

Conception and designed the work, supplied materials, Conceptualization, Methodology, isolated AD-MSCs, Software, Data curation, Writing- Original draft preparation, Supervision, Reviewing, analyzed and interpreted data, final approval of the version to be published.

**Al Shaimaa A. I. Alghriany**

Visualization, Investigation, Assisted with experiments, Validation, Writing, Reviewing, supplied materials, contributed to the study design, isolated AD-MSCs, and wrote histopathology and reference portions of the manuscript.

## Funding

This research did not receive any specific grant from funding agencies in the public, commercial, or not-for-profit sectors.

## Declaration of Competing Interest

The authors declare no competing financial interests.
